# The standardized ileal digestible lysine-to-net energy ratio in the diets of sows to optimize milk nitrogen retention is dynamic during lactation

**DOI:** 10.1093/jas/skae094

**Published:** 2024-04-01

**Authors:** Madelaine C Watzeck, Lee-Anne Huber

**Affiliations:** Department of Animal Biosciences, University of Guelph, Guelph, ON N1G 2W1, Canada; Department of Animal Biosciences, University of Guelph, Guelph, ON N1G 2W1, Canada

**Keywords:** lactation, lysine, net energy, nitrogen utilization, sow

## Abstract

Fifty-two multiparous sows (average parity 3.1 ± 0.9 and initial BW 245.6 ± 32.5 kg) were used to evaluate the effects of dietary standardized ileal digestible (**SID**) Lys-to-net energy (**NE**) ratios on nitrogen (**N**) utilization throughout a 24-d lactation period. Sows were randomly assigned to one of five isoenergetic feeding programs that provided equally spaced and increasing SID Lys-to-NE ratios between 2.79 and 5.50 g SID Lys/Mcal NE. The feeding programs were generated by blending the two extreme diets in varying proportions and were provided to sows immediately after farrowing (day 1) and until weaning at day 24 ± 1. Nitrogen balances were conducted between days 4 and 7, 12 and 15, and 20 and 23 ± 1 of lactation to represent weeks 1, 2, and 3, respectively, using total urine collection and fecal grab sampling. Contrast statements were used to determine the linear and quadratic effects of increasing Lys-to-NE ratios. Linear and quadratic broken-line and polynomial quadratic (**QPM**) models were used to determine the optimum dietary Lys-to-NE ratios for N retention in milk. The Bayesian information criterion was used to assess the best fit. Feeding program did not influence sow average daily feed intake (5.8 ± 0.1 kg), BW change (−8.2 ± 3.1 kg), or change in back fat thickness (−2.6 ± 0.7 mm) over the 24-d lactation period, but piglet average daily gain increased with dietary SID Lys-to-NE ratio (linear; *P* < 0.05). Sow N intake increased with increasing dietary Lys-to-NE ratio in weeks 2 and 3 (linear; *P* < 0.001). Whole-body N retention (N intake − N output in urine and feces) increased with increasing dietary Lys-to-NE ratio in all weeks (linear; *P* < 0.05). The N retention in milk tended to increase then decrease with increasing dietary Lys-to-NE ratio in weeks 1 and 2 (quadratic; *P* = 0.051 and *P* = 0.081) and the QPM showed optimal milk N retention at 4.28, 4.42, and 4.67 g Lys/Mcal NE for weeks 1, 2, and 3, respectively. Maternal N retention (N intake − N output in urine, feces, and milk) decreased and then increased in week 1 (quadratic; *P* < 0.01) and increased in weeks 2 and 3 (linear; *P* < 0.01) with increasing dietary Lys-to-NE ratio. Therefore, the SID Lys-to-NE ratio necessary to optimize milk N output is dynamic throughout lactation. A two-diet feeding program could be created to match optimal weekly or daily SID Lys-to-NE ratios, which could lead to improved piglet ADG and body weights at weaning.

## Teaser

The dietary Lys-to-net energy ratio necessary to optimize milk nitrogen output is dynamic throughout lactation in sows. Optimal weekly ratios could be used to create a precision-feeding program during lactation, which could lead to improved piglet average daily gain and body weights at weaning.

## Introduction

Lactation is a metabolically demanding phase in the reproductive cycle of a sow characterized by a significant increase in nutrient and energy requirements to support both milk production and mammary growth (Feyera and Theil, 2017). Appetite is a major limiting factor during lactation, resulting in energy and protein mobilization from maternal body pools to meet the demands of milk production (Pedersen et al., 2016; Tokach et al., 2019), which can have negative repercussions for the future reproductive performance of the sow (Huang et al., 2013). Overall, it is energetically inefficient for the sow to mobilize body pools to support milk production rather than directly using available energy and amino acids (**AA**) from the diet (Revell et al., 1998; Pedersen et al., 2016; Feyera et al., 2020).

Despite significant changes in nutrient and energy requirements as well as voluntary feed intake during lactation, sows are typically fed a single diet with a static nutrient and energy composition throughout the entire lactation period (Pedersen et al., 2016). Using a factorial approach, Feyera and Theil (2017) postulated that the optimal standardized ileal digestible (**SID**) Lys-to-energy ratio in the diets of sows is dependent on sow milk production and body weight. Considering that milk production changes throughout lactation simultaneously with sow feed intake, the question remains whether a static feeding program is appropriate or if the Lys-to-energy ratio should be adjusted depending on the stage of lactation.

We hypothesized that the optimal ratio of SID Lys-to-net energy (**NE**) for lactating sows will evolve corresponding to changes in milk production as lactation progresses. The objective of the current study was to determine the dietary SID Lys-to-NE ratio that optimized sow milk nitrogen output in each week of lactation.

## Materials and Methods

The experimental protocol was approved by the University of Guelph Animal Care Committee and followed Canadian Council on Animal Care guidelines (CCAC, 2009). The study was conducted at the Arkell Swine Research Station (University of Guelph, Guelph, ON, Canada).

### Animals and housing

Fifty-two multiparous sows (Yorkshire or Yorkshire × Landrace) were recruited to the study over five consecutive breeding batches (blocks). Sows were housed in conventional farrowing crates from day 110 ± 0.6 of gestation and received approximately 2 kg/d of a standard lactation diet. Upon farrowing, the numbers of piglets born alive, stillborn, and mummified were recorded and litters were standardized to 12.7 ± 1.3 piglets within the first 24 h of birth (day 1). After farrowing, sows were supplied the assigned feeding program via a feeder capable of blending two diets (Gestal Quattro, JYGA Technologies, St-Lamert-de-Lauzon, Quebec, Canada). Both piglets and sows had *ad libitum* access to water. Piglets were processed according to the farm protocol (i.e., ear notching, needle teeth clipping, tail docking, and iron injection) within 24 h of birth. Surgical castration was performed on males on day 4 after birth. Creep feed was not supplied to piglets to ensure that piglet BW gain reflected sow milk production. Individual sows and piglets were weighed on days 1, 4, 12, 20, and 24 ± 1 (weaning) after farrowing. In addition, piglets were also weighed on days 7 and 15. Sow backfat (**BF**) depth was measured at the P2 position (6.5 cm from the midline over the last rib) on days 1 ± 1 and 23 ± 1 of lactation using a portable ultrasound machine with a 140 mm linear probe (Agroscan L, ECM Noveko International Inc., Angoulême, France).

### Dietary treatments and feeding

The electronic sow feeders in each farrowing crate were used to blend two basal diets and were calibrated weekly by dispensing 500-g allotments of each diet. The two basal diets were isocaloric (2,543 kcal/kg NE) but were formulated to contain SID Lys-to-NE ratios of 2.79 and 5.50 g SID Lys/Mcal NE and to meet or exceed the AA-to-Lys ratios recommended by the NRC (2012; Table 1). The basal diets were blended at ratios of 75:25, 50:50, and 25:75, respectively, to achieve the equally spaced intermediate Lys-to-NE ratios of 3.47, 4.15, and 4.83 g SID Lys/Mcal NE in addition to the two basal diets for a total of five dietary treatments. The 50:50 blend met the estimated energy and SID Lys requirements for sows using the NRC (2012) lactating sow model with the inputs: average BW after farrowing of 240 kg, average of 13 pigs nursed, and piglet average daily gain (ADG) of 250 g over a 24-d lactation period. Sows were assigned to diets to balance initial BW, parity, and breed and received the same diet for the entire 24-d lactation period. A progressive feeding curve was created to provide approximately 2.5 kg of feed on day 1, 5.0 kg on day 4, 6.4 kg on day 7, and 8.3 kg of feed on day 24 of lactation. The electronic sow feeders released feed in multiple portions when the sensor was stimulated by the sow based on the weight of the total feed allotment for the day, in three feeding periods between 0800 and 1000 hours, 1200 and 1400 hours, and 1600 and 1800 hours. Between days 4 and 7 ± 1, 12 and 15 ± 1, and 20 and 23 ± 1 feed was weighed, blended manually, and provided in three equal meals at 0800, 1200, and 1600 hours to facilitate feed intake estimates during nitrogen balance periods. Feed refusals were monitored daily to calculate average daily feed intake. Titanium dioxide was incorporated into the diets as an indigestible marker to estimate apparent total tract nitrogen digestibility.

**Table 1.** Ingredient composition and nutrient content of experimental diets (as-fed)

	SID Lys:NE, g/Mcal^1^	
	2.79	5.50
Ingredient composition, %		
Corn	47.19	34.52
Soybean meal	9.30	15.00
Soybeans, full fat	10.00	25.00
Wheat, soft red	20.00	20.00
Soybean hulls	6.50	—
Fat, animal-vegetable blend	3.00	—
Monocalcium phosphate	1.60	1.45
Limestone	1.00	1.00
Sodium chloride	0.50	0.50
Premix^2^	0.60	0.60
l-Lys-HCL	0.15	0.55
l-Val	0.04	0.38
l-Thr	0.02	0.24
l-Leu	—	0.19
dl-Met	—	0.15
l-Phe	—	0.14
l-His	—	0.09
l-Ile	—	0.05
l-Trp	—	0.04
Titanium dioxide	0.10	0.10
Total	100.00	100.00
Calculated nutrient content^3^		
Net energy, Kcal/kg	2,554	2,546
Crude protein, %	12.31	17.85
SID Arg, %	0.79	1.22
SID His, %	0.33	0.56
SID Ile, %	0.49	0.79
SID Leu, %	1.06	1.59
SID Lys, %	0.71	1.40
SID Met, %	0.22	0.44
SID Met + Cys, %	0.45	0.75
SID Phe, %	0.61	1.01
SID Phe + Tyr, %	1.00	1.59
SID Thr, %	0.45	0.88
SID Trp, %	0.15	0.27
SID Val, %	0.60	1.12
STTD P, %^4^	0.44	0.45
Fermentable fiber, %	10.93	11.91
Total Ca, %	0.88	0.88
Analyzed nutrient content, %^5^		
Dry matter	87.87	88.92
Crude protein	13.73	19.76
Arg	0.87 (0.88)^6^	1.20 (1.36)
His	0.44 (0.50)	0.60 (0.64)
Ile	0.62 (0.58)	0.90 (0.93)
Leu	1.33 (1.24)	1.79 (1.86)
Lys	0.85 (0.85)	1.47 (1.49)
Met	0.26 (0.26)	0.42 (0.49)
Met + Cys	0.54 (0.55)	0.75 (0.87)
Phe	0.83 (0.71)	1.16 (1.17)
Phe + Tyr	1.21 (1.20)	1.67 (1.86)
Thr	0.57 (0.56)	0.87 (1.04)
Val	0.79 (0.72)	1.21 (1.37)

^1^Standardized ileal digestible Lys-to-NE ratio.

^2^Provided the following amounts of vitamins and trace minerals per kg of premix: vitamin A, 2,000 KIU; vitamin D, 200 KIU; vitamin E, 8,000 IU; vitamin K, 500 mg; thiamin, 300 mg; riboflavin, 1,000 mg; niacin, 5,000 mg; pantothenic acid, 3,000 mg; pyridoxine, 300 mg; choline, 100,000 mg; folacin, 400 mg; biotin, 40 mg; vitamin B12, 5,000 µg; calcium, 22.67% as CaCO_3_; manganese, 4,000 mg as Mn SO_4_·H_2_O; zinc, 21,000 mg as ZnSO_4_; iron, 20,000 mg as FeSO_4_; copper, 3,000 mg as CuSO_4_; selenium, 60 mg as Na_2_SeO_3_; iodine, 100 mg as C_2_H_10_I_2_N_2._ (Grand Valley Fortifiers, Cambridge, ON, Canada).

^3^Based on digestible nutrient and NE contents of feed ingredients according to the NRC (2012).

^4^Standardized total tract digestible.

^5^Analyzed values for composite sample of three batches per diet.

^6^Calculated values are shown in parentheses.

### Nitrogen balance procedure, blood, and milk sampling

Nitrogen balances were conducted on each sow during weeks 1 (between days 4 and 7 ± 1), 2 (between days 12 and 15 ± 1), and 3 of lactation (between days 20 and 23 ± 1). Total urine collection and fecal grab sampling were performed. Briefly, sterile Foley urinary catheters (BARDEX I.C., 2-way, Specialty, Tiemann Model, 75cc balloon, 18FR, Bard Medical, Covington, GA) were lubricated and inserted into the bladder. The balloon was inflated with ~55 mL of saline solution. Polyvinyl tubing was used to connect the catheter to a covered bucket containing 20% sulphuric acid, maintaining a pH of less than 3. Following each successful 24 h collection, urine collection buckets were weighed, and a 5% (wt) subsample was pooled per sow for each nitrogen balance period and stored at 4 °C. If there was an unsuccessful 24 h collection period, one additional day was added to the end of the nitrogen balance period to achieve three full days of collection. Urinary catheters were removed at the end of each nitrogen balance period, pooled urine aliquots were thoroughly mixed and a subsample was collected and stored at 4 °C until further analysis. Fresh feces were collected by manually stimulating the anus daily, pooled per sow for each nitrogen balance period, and frozen at −20°C until further analysis.

On days 4 ± 1, 12 ± 1, and 20 ± 1, sows were fasted for 12 ± 3 hours and 10 mL of blood were collected from each sow via sub-orbital sinus puncture into heparinized tubes (BD vacutainer; Franklin lakes, NJ, USA). Blood samples were centrifuged at 3,000 × *g* at room temperature for 15 min. Plasma samples were aliquoted into microcentrifuge tubes and stored at −20 °C until further analysis.

Milk was collected on days 7 ± 1, 15 ± 1, and 23 ± 1, at the end of each nitrogen balance period. Piglets were removed from the sow for approximately 1 h after which 1 mL of oxytocin preparation was injected intramuscularly into each sow [Oxyto-Sure (20 USP/mL), Vetoquinol, QC, Canada]. Approximately 150 mL of milk were manually collected from one of each anterior and posterior gland until the glands were completely empty. Piglets were then returned to the sow and allowed to suckle. Milk samples were pooled per sow within a day and stored at −20 °C until further analysis.

### Sample analysis

Subsamples of the basal diets (2.79 and 5.50 g SID Lys/Mcal NE) were collected every other week and pooled within batch; a representative composite sample of the three batches was then used for chemical analysis. Fecal samples were freeze-dried and ground to achieve a uniform particle size. Freeze-dried fecal (duplicate) and feed (quadruplicate) samples were analyzed for dry matter and ash contents at 135 °C for 2 h (AOAC, 2005; method 930.15) and then 600 °C for 12 h in a muffle furnace (AOAC, 2005; method 942.05), respectively. Titanium contents in both feces and diets were quantified following the procedure described by Crosbie et al. (2020) and absorbance of standards and samples were measured by spectrophotometry (Epoch 2, BioTek Instruments Inc.Winooski, VT) at 407 nm.

Milk samples were thawed, well mixed, and a 20 mL subsample was weighed into a 50 mL falcon tube prior to freeze drying to determine dry matter (FreeZone, Labconco Corporation, Kansas City, MO). The freeze-dried milk was used to determine the crude fat content using a high-temperature solvent extraction (Ankom, XT29 Fat Analyzer, Macedon, NY, USA; AOCS Official Procedure Am 5-04, 2014). Urinary, feed, and freeze-dried fecal and milk nitrogen (**N**) contents were measured via combustion (LECO-FP 828 analyzer; LECO Instruments Ltd., Mississauga, ON, Canada) and milk crude protein content was calculated as N × 6.38.

Plasma samples were analyzed for AA concentrations using ultra-performance liquid chromatography (adapted from Bidlingmeyer et al., 1984; Waters Corporation, Milford, MA) as described by Banton et al. (2021). All AA except Met and Cys were analyzed following the acid hydrolysis method, while the performic acid oxidation with acid hydrolysis (sodium metabisulfite method) was used for the determination of Met and Cys concentrations (Method 994.12; AOAC, 2005; Waters Corporation). Derivatization was completed using an AccQ-Tag Ultra derivatization kit (Waters Corporation). The obtained AA peak areas were compared with known standards, and the data were analyzed using Waters Empower 2 Software (Waters Corporation). Feed AA contents were analyzed similarly but using oxidation hydrolysis.

### Calculations and statistical analysis

Nitrogen retention was calculated by subtracting the nitrogen excreted daily in urine and feces from nitrogen intake (Möhn and de Lange, 1998). Nitrogen intake was determined from daily feed intake and the analyzed nitrogen contents of the diets, using weighted averages of the basal diet N contents for the intermediate ratio diet blends. Fecal nitrogen output was calculated from nitrogen intake and apparent fecal nitrogen digestibility (Zhu et al., 2005). Sow milk yield was determined following the approach outlined by the NRC (2012). The estimation utilized the 24-d litter growth rate, litter size, and a standard lactation curve (NRC, 2012; Eq. 8-71 and 8-72). Total milk nitrogen output was calculated using the estimated milk yield and the analyzed milk nitrogen value and maternal nitrogen retention was calculated as total nitrogen retention minus milk nitrogen retention. The apparent AA utilization efficiencies for milk production were calculated as described by Huber et al. (2016).

All statistical analyses were conducted using the Proc GLIMMIX function of SAS 9.4 with sow (or litter) as the experimental unit. The model included diet as the fixed effect, while block was considered a random effect. Plasma AA concentrations were log transformed before analysis, and subsequently, the results were back-transformed for presentation. Contrast statements were used to assess the linear and quadratic effects of increasing Lys-to-NE ratios. Probability (***P***) values less than 0.05 were deemed statistically significant, while 0.05 ≤ *P* ≤ 0.10 were considered a trend; *P*-values greater than 0.10 were considered not significant. Linear, quadratic broken-line and polynomial quadratic models (**QPM**), as described by Gonçalves et al. (2016), were used to determine the optimum dietary Lys-to-NE ratios for milk N retention in each week of lactation and piglet ADG over the entire lactation period. The Bayesian information criterion was used to assess the best fit.

## Results

Analyzed dietary crude protein and AA contents were comparable to calculated values (Table 1). Four sows were removed from the study due to farrowing difficulties, illness, prolapse, or lameness, and the data were excluded from the statistical analysis. One sow was removed from the 2.79 g SID Lys/Mcal NE treatment group after successfully completing the N balance in week 1. These data were included in the statistical analysis. Five other sows were removed from specific nitrogen balance periods only due to minor setbacks from which a full recovery was achieved (e.g., farrowing difficulty and/or short-term reduction in feed intake); one sow from the 4.15 g SID Lys/Mcal NE treatment group and four sows from the 4.83 group did not participate in the first N balance. In total, 47 sows completed the entire 24 ± 1-d lactation period.

**Figure 1.**
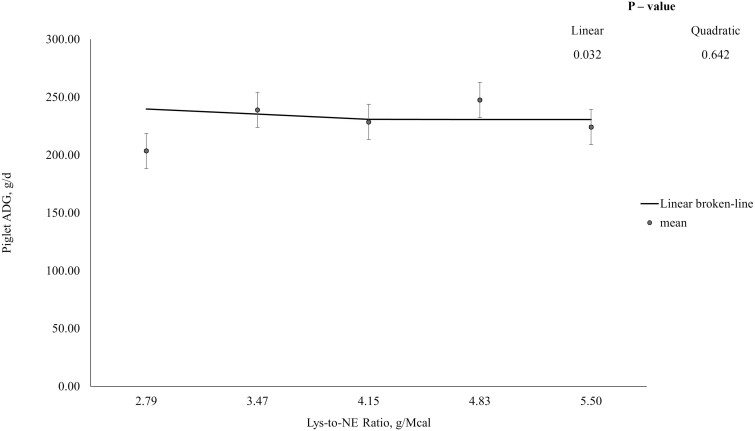
Piglet average daily gain (ADG) over a 24-d lactation period in response to the standardized ileal digestible (SID) Lys-to-net energy (NE) ratio. Data were best described by the quadratic polynomial model and the optimum SID Lys-to-NE ratio was identified at 4.3 g Lys/Mcal NE. Piglet ADG, g/d = 102.4 + 64.51 × (SID Lys-to-NE ratio)—7.5 × (SID Lys-to-NE ratio)^2^.

Feeding program did not influence sow body weight change, change in backfat thickness, or ADFI over the entire 24-d lactation period, nor litter size at weaning (Table 2). However, piglet BW at weaning and overall piglet ADG increased with dietary SID Lys-to-NE ratio (linear; *P* < 0.05). The QPM had the best fit for overall piglet ADG and an optimum SID Lys-to-NE ratio was identified at 4.3 g Lys/Mcal NE (Figure 1), which corresponded to 63.6 g/d SID Lys intake.

**Table 2.** Sow and litter growth performance over a 24-d lactation for sows fed one of five isoenergetic feeding programs that provided equally spaced and increasing standardized ileal digestible (SID) Lys-to-NE ratios

Item	Diet^1^					SEM^2^	*P*-value^3^	
	2.79	3.47	4.15	4.83	5.50		Linear	Quadratic
No. of sows	10	9	9	10	9			
Average parity	3.3 ± 0.6	3.1 ± 0.9	3.1 ± 1.1	3 ± 1.1	2.9 ± 0.8			
Litter size at farrowing^4^	12.9	12.9	12.8	12.7	12.4	0.5	0.652	0.936
Litter size at weaning	11.4	11.2	11.1	11.4	11.3	0.4	0.998	0.594
Initial average piglet weight, kg	1.5	1.4	1.5	1.5	1.4	0.1	0.756	0.632
Final average piglet weight, kg	6.1	6.8	6.7	7.1	6.5	0.3	0.040	0.610
Piglet average daily gain, g	206	239	229	250	226	14	0.032	0.642
Initial sow body BW, kg	250.6	249.4	245.2	251.4	236.6	11.5	0.970	0.720
Change in sow BW, kg	−8.4	−6.5	−4.2	−13.4	−9.6	3.5	0.375	0.117
Initial sow back fat, mm	14.8	14.2	16.8	14.2	15.2	1.3	0.903	0.376
Change in sow back fat, mm	−2.0	−3.3	−2.3	−2.1	−3.4	1.0	0.891	0.377
ADFI, kg, as-fed^5^	5.9	5.7	6.0	5.7	5.8	0.2	0.532	0.972

^1^Increasing SID Lys-to-NE ratios, g/Mcal.

^2^SEM based on repeated measures analysis (largest value across treatments).

^3^Probability values for linear and quadratic contrasts.

^4^Litter size after standardization.

^5^Sow average daily feed intake.

**Figure 2.**
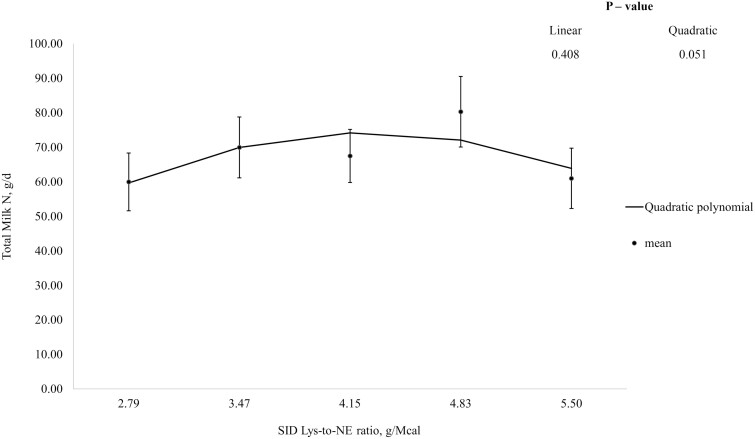
Total daily milk nitrogen (N) yield between days 4 and 7 ± 1 (week 1) of lactation. Data were best described by the quadratic polynomial model and the optimum SID Lys-to-NE ratio was identified at 4.28 g SID Lys/Mcal NE. Total Milk N, g/d = −48.07 + 57.39 × (SID Lys-to-NE ratio) − 6.73 × (SID Lys-to-NE ratio)^2^.

**Figure 3.**
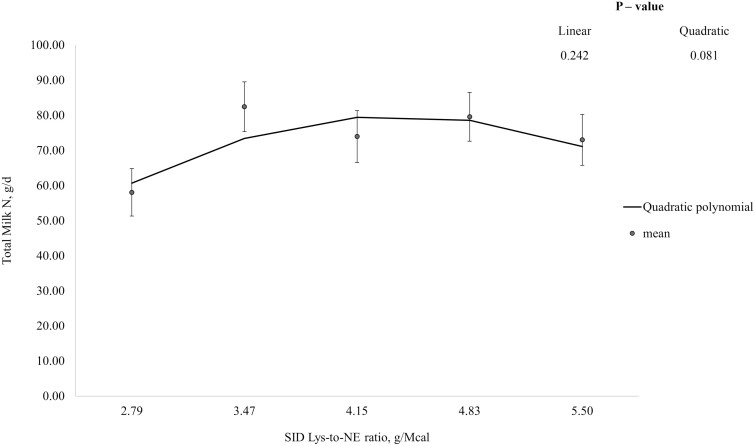
Total daily milk nitrogen (N) yield between days 12 and 15 ± 1 (week 2) of lactation. Data were best described by the quadratic polynomial model and the optimum SID Lys-to-NE ratio was identified at 4.42 g SID Lys/Mcal NE. Total Milk N, g/d = −63.12 + 64.87 × (SID Lys-to-NE ratio) − 7.35 × (SID Lys-to-NE ratio)^2^.

**Figure 4.**
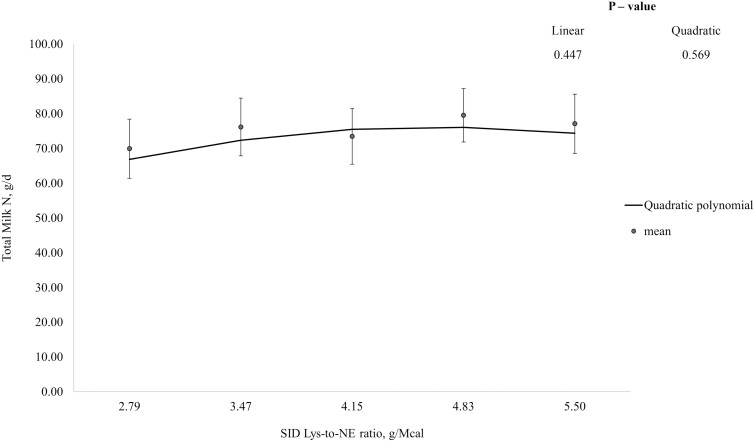
Total daily milk nitrogen (N) yield between days 20 and 23 ± 1 (week 3) of lactation. Data were best described by the quadratic polynomial model and the optimum SID Lys-to-NE ratio was identified at 4.67 g SID Lys/Mcal NE. Total Milk N, g/d = 18.71 + 24.60 × (SID Lys-to-NE ratio) − 2.63 × (SID Lys-to-NE ratio)^2^.

In week 1, N intake tended to increase (linear; *P* = 0.079), and total N excretion, N excretion in feces and urine, N absorbed, and N retention increased (linear; *P* < 0.05) with increasing dietary Lys-to-NE ratio, but apparent N retention efficiencies were not influenced by dietary treatment (Table 3). Total milk N output tended to increase then decrease (quadratic; *P* = 0.051) and maternal N retention decreased then increased (quadratic; *P* < 0.05) with increasing Lys-to-NE ratio. In week 2, N intake, N absorbed, N retained, and maternal N retention increased with increasing dietary Lys-to-NE ratio (linear; *P* < 0.001) but N excretion, and apparent N retention efficiency were not influenced by dietary treatment. Total milk N output tended to increase then decrease with increasing Lys-to-NE ratio (quadratic; *P* = 0.081). In week 3, N intake, total and urinary N excretion, N absorbed, N retained, and maternal N retention increased with dietary Lys-to-NE ratio (linear; *P* < 0.001) but fecal N excretion, total milk N output, and apparent N retention efficiency (% of intake) were not influenced by dietary treatment. Apparent N retention efficiency (% of absorbed) tended to decrease with increasing Lys-to-NE ratio (linear; *P* = 0.075). The QPM had the best fit for total milk N output and optimum Lys-to-NE ratios were identified at 4.26, 4.42, and 4.67 g Lys/Mcal NE for weeks 1, 2, and 3 of lactation, respectively (Figures 2, 3, and 4, respectively), which corresponded to SID Lys intakes of approximately 50.2, 80.6, and 95.5 g/d, respectively.

**Table 3.** Nitrogen utilization between days 4 and 7 ± 1 (week 1), days 12 and 15 ± 1 (week 2), and days 20 and 23 ± 1 (week 3) of lactation in sows fed one of five isoenergetic feeding programs that provided equally spaced and increasing standardized ileal digestible (SID) Lys-to-NE ratios

Item	Diet^1^					SEM^2^	*P*-value^3^	
	2.79	3.47	4.15	4.83	5.50		Linear	Quadratic
Week 1 (days 4 to 7 ± 1)								
No. of sows	11	9	8	6	9			
Average parity	3.3 ± 0.6	3.2 ± 0.9	3.0 ± 1.1	2.9 ± 1.1	2.9 ± 0.8			
N intake, g/d	125.5	122.5	145.2	141.1	178.9	9.8	0.079	0.943
Total N excretion, g/d	57.7	53.8	63.3	72.9	80.6	8.1	0.004	0.358
Fecal N excretion, g/d	20.3	20.7	19.7	23.4	26.6	2.8	0.040	0.242
Urine N excretion, g/d	37.3	33.1	43.5	49.5	54.0	7.0	0.008	0.549
N absorbed, g/d	105.1	101.8	125.5	117.6	152.3	9.4	<0.001	0.136
N retention, g/d	65.4	68.7	81.9	68.2	98.3	10.8	0.024	0.433
Total milk N, g/d^4^	59.9	71.1	68.4	81.3	62.5	8.1	0.408	0.051
Maternal N retention, g/d	17.5	−2.7	12.9	−12.8	28.0	12.8	0.733	0.042
N retained, % of intake	53.4	54.6	55.5	47.1	55.0	6.4	0.798	0.829
N retained, % of absorbed	62.7	65.6	63.8	56.8	64.5	7.3	0.776	0.846
Week 2 (days 12 to 15 ± 1)								
No. of sows	10	9	9	10	9			
Average parity	3.4 ± 0.8	3.1 ± 0.9	3.1 ± 1.0	3 ± 1.1	2.9 ± 0.8			
N intake, g/d	185.4	196.5	219.1	234.6	257.4	110.3	<0.001	0.766
Total N excretion, g/d	71.5	66.3	85.5	77.9	80.6	6.6	0.132	0.645
Fecal N excretion, g/d	27.0	25.4	27.5	24.1	27.3	1.7	0.865	0.442
Urine N excretion, g/d	44.8	40.5	57.5	53.3	53.3	6.2	0.106	0.572
N absorbed, g/d	158.4	171.2	191.6	210.6	230.1	9.1	<0.001	0.629
N retention, g/d	113.7	132.6	134.8	158.3	177.1	9.4	<0.001	0.466
Total milk N, g/d	58.7	80.9	72.6	79.1	71.7	7.1	0.242	0.081
Maternal N retention, g/d	55.6	52.3	62.7	79.6	105.5	12.2	<0.001	0.112
N retained, % of intake	62.0	68.1	61.4	67.3	67.5	2.6	0.196	0.945
N retained, % of absorbed	72.0	77.2	70.3	74.9	76.3	3.1	0.474	0.724
Week 3 (days 20 to 23 ± 1)								
No. of sows	10	9	9	10	9			
Average parity	3.0 ± 0.7	3.2 ± 0.8	3.1 ± 1.1	3 ± 1.1	2.9 ± 0.8			
N intake, g/d	199.2	224.1	242.6	267.0	281.7	7.1	<0.001	0.967
Total N excretion, g/d	72.0	72.4	87.7	87.6	108.4	5.1	<0.001	0.176
Fecal N excretion, g/d	30.3	28.4	30.3	30.5	32.6	2.1	0.285	0.404
Urine N excretion, g/d	41.6	43.4	57.0	57.0	75.4	5.1	<0.001	0.274
N absorbed, g/d	169.1	195.7	212.5	236.5	249.1	6.2	<0.001	0.311
N retention, g/d	127.3	153.1	155.7	179.5	174.3	9.3	<0.001	0.161
Total milk N, g/d	66.2	75.7	72.0	77.9	74.3	7.4	0.447	0.569
Maternal N retention, g/d	59.3	75.8	80.5	100.9	98.4	10.9	<0.001	0.498
N retained, % of intake	64.0	68.0	64.1	67.3	60.9	2.5	0.324	0.102
N retained, % of absorbed	75.4	77.9	73.1	75.9	69.2	2.8	0.075	0.256

^1^Increasing SID Lys-to-NE ratios, g/Mcal.

^2^SEM based on repeated measures analysis (largest value across treatments).

^3^Probability values for linear and quadratic contrasts.

^4^Calculated using litter size and piglet average daily gain between days 4 and 7 for week 1, days 12 and 15 for week 2, and days 20 and 23 for week 3 and analyzed milk N concentration on days 7, 15, and 21, respectively.

On days 7, 15, and 23, estimated milk yield and chemical composition (i.e., dry matter, crude fat, and crude protein concentrations) were not influenced by dietary treatment (Table 4). On day 4, plasma concentrations of Thr, total essential AA (**EAA**), Cys, and Tyr decreased then increased (quadratic; *P* < 0.05), plasma concentrations of Phe, Trp, and Val tended to decrease then increase (quadratic; *P* = 0.063, 0.065, and 0.056, respectively), plasma concentrations of Ala, Glu, and Pro decreased (linear; *P* < 0.05), and plasma concentrations of His tended to decrease (linear; *P* = 0.080) with increasing dietary Lys-to-NE ratio. On day 12, plasma concentrations of Val and total EAA increased (linear; *P* < 0.001 and *P* = 0.076, respectively), Glu, Gln, and Gly decreased (linear; *P* < 0.05), Ser and total nonessential AA (**NEAA**) tended to decrease (linear; *P* = 0.055 and 0.056, respectively), and Tyr decreased then increased (quadratic; *P* < 0.05) with increasing Lys-to-NE ratio. On day 20, plasma concentrations of Val and Lys increased (linear; *P* < 0.001 and *P* = 0.051, respectively), Ala, Glu, and total NEAA decreased (linear; *P* < 0.05), Gly tended to decrease (linear; *P* = 0.063), and Cys increased then decreased (quadratic; *P* < 0.05) with increasing Lys-to-NE ratio (Table 5).

**Table 4.** Milk composition for sows fed one of five isoenergetic feeding programs that provided equally spaced and increasing standardized ileal digestible (SID) Lys-to-NE ratios

Item	Diet^1^					SEM^2^	*P*-value^3^		
	2.79	3.47	4.15	4.83	5.50		Diet	Linear	Quadratic
Day 7 ± 1									
No. of sows	11	9	8	6	9				
Average parity	3.3 ± 0.6	3.2 ± 0.9	3 ± 1.1	2.9 ± 1.1	2.9 ± 0.8				
Dry matter, %	18.6	18.4	19.7	20.9	18.9	0.8	0.125	0.165	0.165
Crude fat, %	7.8	7.3	8.4	9.0	7.8	0.6	0.178	0.268	0.299
Crude protein, %^4^	5.2	5.5	5.4	5.7	5.2	0.3	0.556	0.785	0.186
Estimated milk yield, kg/d	7.2	8.6	8.1	9.0	7.6	0.8	0.276	0.509	0.092
Day 15 ± 1									
No. of sows	10	9	10	10	9				
Average parity	3.3 ± 0.6	3.1 ± 0.9	3.1 ± 1.0	3.0 ± 1.1	2.9 ± 0.8				
Dry matter, %	19.4	20.5	19.4	19.3	19.4	1.2	0.906	0.649	0.772
Crude fat, %	7.7	7.7	8.4	8.5	7.9	0.7	0.708	0.503	0.338
Crude protein, %	5.1	5.3	5.1	5.0	5.4	0.3	0.714	0.858	0.540
Estimated milk yield, kg/d	8.3	9.5	8.5	10.3	8.5	0.8	0.275	0.583	0.266
Day 23 ± 1									
No. of sows	10	9	9	10	9				
Average parity	3.3 ± 0.6	3.1 ± 0.9	3.1 ± 1.1	3.0 ± 1.1	2.9 ± 0.8				
Dry matter, %	19.0	18.0	19.7	19.0	19.1	0.8	0.547	0.557	0.989
Crude fat, %	7.6	6.3	8.3	7.4	7.0	0.7	0.244	0.930	0.645
Crude protein, %	5.3	5.2	5.8	5.4	5.7	0.2	0.246	0.118	0.763
Estimated milk yield, kg/d	7.9	9.3	8.0	9.2	8.2	0.8	0.514	0.814	0.393

^1^Increasing SID Lys-to-NE ratios, g/Mcal.

^2^SEM based on repeated measures analysis (largest value across treatments).

^3^Probability values for linear and quadratic contrasts.

^4^Milk crude protein was estimated as milk N × 6.38.

**Table 5.** Post-absorptive plasma concentration of essential and nonessential amino acids on days 4, 12, and 20 of lactation for sows fed one of five isoenergetic feeding programs that provided equally spaced and increasing standardized ileal digestible (SID) Lys-to-NE ratios

Item	Diet^1^					SEM^2^	*P*-value^3^	
	2.79	3.47	4.15	4.83	5.5		Linear	Quadratic
Day 4 ± 1								
No. of sows	9	9	7	9	9			
Average parity	3.3 ± 0.6	3.2 ± 0.9	3.0 ± 1.1	3.0 ± 1.1	2.9 ± 0.8			
EAA^4^, μmol/L								
Arg	132	107	96	113	118	16	0.649	0.102
His	98	95	83	81	84	11	0.080	0.480
Ile	105	86	96	88	97	14	0.784	0.397
Leu	165	134	145	101	141	19	0.122	0.174
Lys	151	124	137	118	132	23	0.435	0.295
Met	54	49	45	49	49	6	0.500	0.280
Phe	69	56	60	53	66	7	0.627	0.063
Thr	135	91	95	114	125	18	0.939	0.031
Trp	37	39	29	32	42	6	0.883	0.065
Val	282	248	256	232	332	37	0.381	0.056
Total EAA	1,171	933	1,009	937	1,194	118	0.875	0.034
NEAA^5^, μmol/L								
Ala	501	546	332	328	357	58	0.021	0.201
Cys	40	29	12	18	31	7	0.114	0.003
Glu	260	202	191	179	190	28	0.034	0.088
Gln	609	525	497	490	516	66	0.218	0.272
Gly	801	763	646	755	655	96	0.200	0.718
Pro	291	239	215	195	204	32	0.011	0.202
Ser	120	120	104	110	116	12	0.569	0.387
Tyr	66	50	54	56	71	8	0.486	0.020
Total NEAA	2,396	2,193	2,053	1,925	2,114	345	0.304	0.423
Day 12 ± 1								
No. of sows	10	10	10	10	8			
Average parity	3.2 ± 0.8	3.2 ± 0.8	3.1 ± 1.0	3.0 ± 1.1	2.9 ± 0.8			
EAA, μmol/L								
Arg	96	96	88	79	100	11	0.685	0.102
His	100	90	76	97	90	11	0.641	0.215
Ile	85	88	81	95	111	12	0.101	0.257
Leu	135	138	140	147	139	19	0.731	0.776
Lys	74	93	79	82	112	20	0.120	0.483
Met	46	49	43	44	51	8	0.578	0.249
Phe	59	56	50	53	63	8	0.840	0.132
Thr	80	73	74	79	94	14	0.295	0.207
Trp	39	39	40	35	42	6	0.893	0.604
Val	201	221	233	256	356	43	<0.001	0.166
Total EAA	886	953	868	955	1,137	116	0.076	0.229
NEAA, μmol/L								
Ala	344	340	319	289	313	44	0.257	0.691
Cys	36	18	37	48	29	13	0.216	0.513
Glu	249	216	205	174	195	26	0.033	0.276
Gln	595	538	419	391	482	53	0.014	0.030
Gly	810	85	676	613	672	100	0.022	0.561
Pro	195	207	202	186	221	29	0.617	0.632
Ser	112	109	97	92	100	10	0.055	0.285
Tyr	59	54	43	52	63	9	0.749	0.036
Total NEAA	1,570	1,438	1,307	1,223	1,382	131	0.056	0.086
Day 20 ± 1								
No. of sows	10	8	9	7	9			
Average parity	3.3 ± 0.6	3.1 ± 0.9	3.1 ± 1.0	3.0 ± 1.1	2.9 ± 0.8			
EAA, μmol/L								
Arg	88	79	85	94	89	11	0.504	0.696
His	93	84	75	79	80	12	0.358	0.419
Ile	88	77	105	104	97	12	0.153	0.564
Leu	153	121	166	139	131	17	0.621	0.673
Lys	70	71	90	98	93	18	0.051	0.599
Met	39	45	48	53	41	9	0.516	0.151
Phe	73	52	63	53	59	9	0.253	0.268
Thr	85	77	85	92	88	12	0.525	0.814
Trp	46	40	45	40	44	7	0.858	0.616
Val	209	197	251	303	302	32	<0.001	0.807
Total EAA	931	818	988	1,002	942	100	0.400	0.866
NEAA, μmol/L								
Ala	355	311	241	338	239	36	0.046	0.627
Cys	23	61	33	30	19	12	0.110	0.022
Glu	252	200	174	220	166	27	0.049	0.451
Gln	507	514	423	560	380	53	0.153	0.388
Gly	765	821	527	829	578	68	0.063	0.694
Pro	198	169	167	204	153	19	0.328	0.916
Ser	111	106	101	107	97	11	0.293	0.938
Tyr	63	48	45	49	44	9	0.159	0.379
Total NEAA	2,259	2,185	1,712	2,327	1,650	173	0.033	0.839

^1^Increasing SID Lys-to-NE ratios, g/Mcal.

^2^SEM based on repeated measures analysis (largest value across treatments).

^3^Probability values for linear and quadratic contrasts.

^4^EAA: essential amino acids; Arg is included as a conditionally EAA.

^5^NEAA: nonessential amino acids.

In weeks 1 and 3, the apparent utilization efficiency of all EAA and N for milk production, except for Arg and His, decreased with increasing dietary Lys-to-NE ratio (linear; *P* < 0.05). In week 2, the apparent utilization efficiencies of Ile, Lys, Met, Phe, Thr, Val, and N decreased with increasing ratio of Lys-to-NE (linear; *P* < 0.05; Table 6).

**Table 6.** Apparent utilization efficiency of dietary amino acids for milk protein production between days 4 and 7 ± 1 (week 1), days 12 and 15 ± 1 (week 2), and days 20 and 23 ± 1 (week 3) of lactation in sows fed one of five isoenergetic feeding programs that provided equally spaced and increasing standardized ileal digestible (SID) Lys-to-NE ratios

Item	Diet^1^					SEM^2^	*P*-value^3^	
	2.79	3.47	4.15	4.83	5.5		Linear	Quadratic
Week 1 (days 4 to 7 ± 1)								
No. of sows	11	9	9	10	9			
Average parity	3.3 ± 0.6	3.2 ± 0.9	3.1 ± 1.0	3.0 ± 1.1	2.9 ± 0.8			
Arg	44.9	44.7	47.9	38.2	36.6	7.7	0.283	0.535
His	57.3	60.1	59.7	53.5	44.3	8.2	0.145	0.260
Ile	56.9	60.9	53.5	48.1	37.1	5.8	0.005	0.189
Leu	54.8	62.7	55.4	52.7	41.8	5.7	0.042	0.117
Lys	70.3	70.0	59.7	51.1	40.0	6.7	<0.001	0.419
Met	61.4	64.8	57.1	48.6	38.1	7.8	0.004	0.253
Met + Cys	60.2	69.0	60.8	56.5	45.1	7.4	0.044	0.143
Phe	44.9	53.2	40.4	46.6	34.0	4.6	0.039	0.159
Phe + Tyr	58.8	67.9	59.9	57.2	44.9	6.4	0.052	0.112
Thr	71.4	77.3	65.8	59.0	45.9	8.0	0.004	0.223
Val	57.1	60.0	52.6	46.1	36.5	6.5	0.006	0.287
N	58.1	60.8	56.3	49.2	40.2	6.3	0.018	0.264
Week 2 (days 12 to 15 ± 1)								
No. of sows	10	9	9	10	9			
Average parity	3.3 ± 0.6	3.1 ± 0.9	3.1 ± 1.0	3.0 ± 1.1	2.9 ± 0.8			
Arg	32.8	35.2	27.0	32.6	25.1	4.9	0.245	0.753
His	41.9	44.7	35.2	42.7	32.6	5.2	0.203	0.636
Ile	43.2	44.5	33.8	38.1	28.9	4.4	0.015	0.712
Leu	41.7	45.0	36.0	41.5	32.9	4.2	0.114	0.542
Lys	54.6	53.0	39.1	42.2	32.6	5.4	0.002	0.955
Met	44.9	45.0	33.7	38.5	27.6	5.0	0.011	0.743
Met + Cys	43.8	46.6	37.0	43.8	33.8	4.8	0.131	0.595
Phe	33.6	35.6	29.3	32.8	25.0	2.8	0.025	0.332
Phe + Tyr	45.5	47.3	37.6	43.8	33.7	4.5	0.105	0.473
Thr	52.3	52.7	40.5	46.0	34.4	5.3	0.013	0.745
Val	41.8	41.7	32.2	36.9	28.0	4.4	0.022	0.833
N	44.9	46.8	36.0	40.8	32.2	5.0	0.047	0.764
Week 3 (days 20 to 23 ± 1)								
No. of sows	10	9	9	10	9			
Average parity	3.2 ± 0.6	3.1 ± 0.9	3.1 ± 1.1	3.0 ± 1.1	2.9 ± 0.8			
Arg	38.6	42.9	37.3	35.0	29.2	6.1	0.166	0.458
His	46.4	51.1	43.5	42.4	36.1	6.1	0.116	0.482
Ile	46.3	47.0	39.3	36.7	30.1	4.8	0.006	0.588
Leu	44.4	46.8	40.4	39.1	33.0	4.6	0.037	0.481
Lys	58.4	57.8	43.0	41.7	34.4	5.8	0.001	0.998
Met	50.4	51.5	41.3	38.4	30.3	5.7	0.004	0.594
Met + Cys	47.7	51.2	42.9	41.8	35.3	5.4	0.037	0.501
Phe	32.6	32.1	26.0	26.4	22.4	2.7	<0.001	0.930
Phe + Tyr	47.4	49.6	42.2	41.3	34.3	4.9	0.027	0.505
Thr	57.6	57.5	46.7	44.1	35.8	5.7	0.002	0.697
Val	47.1	47.4	38.5	36.3	29.6	5.0	0.005	0.700
N	48.9	51.3	44.0	41.0	35.0	5.9	0.044	0.573

^1^Increasing SID Lys-to-NE ratios, g/Mcal.

^2^SEM based on repeated measures analysis (largest value across treatments).

^3^Probability values for linear and quadratic contrasts.

## Discussion

The overall goal of this study was to determine the dietary SID Lys-to-NE ratio that optimized sow milk N output in each week of lactation. Based on the QPM analyses, it appears that the Lys-to-NE ratio required to optimize N output in milk is dynamic throughout lactation (4.26, 4.42, and 4.67 g SID Lys/Mcal NE in lactation weeks 1, 2, and 3, respectively). In addition, litter growth rate was driven by milk yield in the absence of creep feed provision, since milk composition was not influenced by the Lys:E ratio at any point during the lactation period. Therefore, using a phase- or precision-feeding program in lactation to offer dynamic Lys-to-NE ratios could be a means to improve milk N output and litter growth. Indeed, previous work has demonstrated that a two-diet feeding approach with SID Lys-to-metabolizable energy ratios 103%, 109%, 110%, and 110% greater than those in a fixed diet for each respective week of a 4-wk lactation led to improved sow milk production, increased piglet ADG, and reduced sow BW loss during the initial stages of lactation when compared to a static feeding program (Pedersen et al., 2016). In the aforementioned study, however, the SID Lys-to-metabolizable energy ratios used were below the range considered in the current study, which could inflate the apparent potential of phase-feeding. In the current study, piglet ADG (i.e., over the entire 24-d lactation period) was optimized at 4.3 g SID Lys/Mcal NE (63.6 g/d SID Lys), which is comparable to current feeding recommendations (e.g., between 3.6 and 4.2 g SID Lys/Mcal NE per current genetics manuals) and those proposed recently by other researchers (i.e., 56.4 g/d SID Lys; as reviewed by Theil et al., 2023). Therefore, the Lys-to-NE ratios selected for the current study were valid but assessing overall piglet ADG does not reflect the dynamic nature of the Lys-to-NE ratios to optimize milk N output. In addition, it is noted that others have shown increased energy and SID Lys supply in the last week prior to farrowing positively influenced milk production in the subsequent lactation period (Bruun et al., 2023), which could alter Lys-to-NE ratios required to optimize (weekly) milk production in the subsequent lactation period. Therefore, further work is required to assess the interaction between pre- and post-farrowing feeding programs.

Sow N intake increased with increasing Lys-to-NE ratio, since whole protein sources and crystalline AA were used to increase Lys supply, while total and milk N retention also increased. Thus, the apparent efficiency of retaining dietary N remained unaffected. The amount of N absorbed and total N excretion both increased with increasing Lys-to-NE ratios (i.e., increasing crude protein levels), as demonstrated in previous studies using lactating sows (King et al., 1993; Huber et al., 2015; Zhang et al., 2019). Despite improvements in milk N output with increasing Lys-to-NE ratio, maternal N retention (total N retention − milk N output) was positive and increased with increasing Lys-to-NE ratio, particularly in lactation weeks 2 and 3 and when feed intake was high. These data are supported by the plasma NEAA concentrations, which decreased with increasing SID Lys-to-NE ratio, indicating a reduction in maternal protein mobilization. Indeed, others have shown that increases in plasma concentrations of Ala, Gly, Gln, and Ser are markers related to AA mobilization from maternal body protein pools (Reynolds et al., 1994).

The increase in maternal N retention with Lys-to-NE ratio could be either beneficial or detrimental. On one hand, the recuperation of maternal protein losses that occurred in late gestation and week 1 of lactation when feed intakes were low, is beneficial for the future productivity and longevity of sows (Dourmad et al., 1994). Conversely, since these sows were parity 3 (average), animals should be approaching mature protein mass and any additional maternal protein deposition would lead to greater maintenance requirements in subsequent reproductive cycles. It is not known whether sows used in the current study mobilized extensive amounts of maternal protein prior to farrowing, though previous data from this herd demonstrated significant reductions in sow loin depth in late gestation using the same farm-standard gestation feeding program as employed prior to the current study (Stewart et al., 2021). Regardless, sows in the current study lost between 4 and 13 kg of BW over the entire 24-d lactation period, demonstrating a misalignment of (maternal) N retention with the sow BW change data, which has also occurred in previous studies (e.g., Huber et al., 2015; Feyera et al., 2020). To some degree, the discrepancy could be due to an overestimation of N retention in N balance studies due to N volatilization from urine despite acidification of the collection vessels. It is also possible that the chemical composition of the maternal body changed over lactation, whereby lipid loss was replaced by protein gain. Increased rates of protein synthesis either in the milk or maternal protein pools would require additional ATP, which can be supplied by mobilization of maternal lipid pools. Indeed, other researchers have suggested that nutrient repartitioning toward milk production likely occurs at the cost of maternal adipose rather than protein tissue (Bergsma et al., 2009; Strathe et al., 2017; Zhang et al., 2020), while oversupply of dietary Leu, in particular, induces nutrient repartitioning away from the mammary gland (Zang et al., 2020). In the current study, the dietary Leu:Lys ratio was 1.31× greater in the low vs. the high Lys-to-NE ratio diet, though almost all essential AA:Lys ratios were inflated in the low Lys-to-NE ratio diet since SID Lys was supplied below estimated requirements. Others have shown significant uptake of branched-chain AA by the mammary gland (Trottier et al., 1997) and that excess Leu can interfere with Lys uptake by mammary tissue (Hurley et al., 2000), which could have reduced milk production by sows that received the lowest Lys-to-NE ratio diets. Conversely, the absolute supply of dietary Leu (vs. the Leu:Lys ratio) could contribute to maternal protein deposition for sows that received the higher Lys-to-NE ratio diets. Directly assessing the changes in sow chemical body composition was beyond the scope of the current study, but based on the loss of backfat depth coupled with positive maternal N retention, it is likely that mobilization of maternal adipose tissue occurred to support increased protein retention in maternal and milk pools with increasing SID Lys-to-NE ratio.

In the current study, the apparent utilization efficiency of most AA for milk production was lower than the biological maximum efficiency values reported by the NRC (2012) for groups of sows. The exception was for Lys only in week 1 where, for the lowest two Lys-to-NE ratios (i.e., 2.79 and 3.47 g SID Lys/Mcal NE), the apparent utilization efficiency was relatively greater than the biological maximum for groups of sows (i.e., 67%; NRC, 2012). Thus, in early lactation, when feed intake was low, the SID Lys supply was limiting milk production, as intended. By weeks 2 and 3, however, when feed intake was approximately 35% greater (vs. week 1), SID Lys intake no longer limited milk production since the apparent utilization efficiencies became less than the biological maximum for groups of sows and AA in excess of the needs for milk production could be used for maternal protein retention. Since SID Lys intake and milk yield are the main drivers of the apparent AA utilization efficiency calculations, the seeming increase in AA partitioning toward maternal N (protein) retention did not influence apparent AA utilization efficiency values calculated in the current study.

In this present research, increasing Lys-to-NE ratios were achieved by adding whole protein sources, crystalline Lys, and by including other crystalline AA to maintain AA:Lys ratios (i.e., L-His, Ile, Leu, Val, and Phe), which reduced apparent N and AA utilization efficiencies for milk production and increased N excretion to the environment. The greater supply and loss of N represents an environmental concern and economic and energetic inefficiencies. Whether implementing a feeding program that supplies dynamic and increasing Lys-to-NE ratios throughout lactation via the addition of whole protein sources results in sufficient improvements in piglet ADG (or piglet BW at weaning) to offset the environmental and economic costs will depend on the management and costing structures of individual systems. Reducing crude protein via the use of crystalline AA to alter the dietary Lys-to-NE ratio could be an alternative formulation approach but further work is required to assess the ideal AA:Lys profiles for lactating sows.

## Conclusion

In conclusion, the SID Lys-to-NE ratio necessary to optimize milk N output is dynamic throughout lactation for sows. Therefore, it is possible to create a two-diet feeding program that provides uniquely blended Lys-to-NE ratios for each week of lactation, which could lead to improved piglet ADG and BW at weaning.

## Abbreviations

AA: amino acids
BF: backfat
EAA: essential amino acids
N: nitrogen
NE: net energy
NEAA: nonessential amino acids
P: probability
QPM: polynomial quadratic models
SID: standardized ileal digestible
